# A Clinically Oriented Framework for Real-Time Heart Rate Variability Analysis: A Novel Approach To Personalized and Robust Monitoring

**DOI:** 10.1007/s10916-026-02342-z

**Published:** 2026-01-29

**Authors:** Takashi Nakano, Masayuki Fujino, Masafumi Miyata, Tetsushi Yoshikawa

**Affiliations:** 1 Department of Computational Biology, School of Medicine, Fujita Health University, Toyoake, Japan; 2 International Center for Brain Science (ICBS), Fujita Health University, Toyoake, Japan; 3 Department of Pediatrics, School of Medicine, Fujita Health University, Toyoake, Japan

**Keywords:** Heart rate variability, Real-time analysis, Adaptive alert, Software, Electrocardiogram

## Abstract

Heart rate variability (HRV) is a well-established, noninvasive measure of autonomic nervous system activity and is associated with clinical outcomes. Although real-time monitoring of HRV is valuable in clinical practice, its effectiveness is often compromised by major challenges: high inter-individual variability and frequent data contamination from procedural artifacts. To address these challenges, we developed and validated a computational framework for robust and personalized real-time HRV analysis oriented toward clinical application. The framework performs simultaneous analysis and visualization of both time- and frequency-domain HRV indices and incorporates an adaptive alert algorithm that personalizes alert thresholds using the interquartile range of each patient’s own data. A workflow-integrated mechanism for manually annotating and excluding artifact-prone periods prevents procedural artifacts from skewing the statistical baselines, and a multi-scale visualization module provides a unified view of short-term fluctuations and long-term trends. While existing HRV tools are powerful for research or offline analysis, they often lack the integration of personalized alerting and workflow-oriented artifact management needed for bedside care. The proposed system uniquely combines personalized alerting, care-linked artifact exclusion, and multi-scale bedside visualization within a single real-time software package. The framework was validated using open-access electrocardiogram (ECG) databases and synthetic noise-contaminated signals, confirming robust R-wave detection across pediatric and adult recordings and under low signal-to-noise conditions. In addition, the framework was operationally validated at the bedside using ECG data from 24 newborn patients. By systematically addressing the core challenges of personalization and artifact management in a clinically integrated manner, this work represents a significant step toward translating real-time HRV analysis into routine vital sign management and, ultimately, improved patient outcomes.

**Supplementary Information:**

The online version contains supplementary material available at 10.1007/s10916-026-02342-z.

## Introduction

Real-time detection of minute changes in circulatory dynamics is crucial in the management of critically ill patients, such as newborns, children and older adults, who are at high risk for sudden deterioration. Early detection of such changes directly improves outcomes, as demonstrated in neonatal care [1]. Heart rate variability (HRV), the beat-to-beat variability in cardiac cycles measured from the electrocardiogram (ECG), is widely recognized as a quantitative, noninvasive method for assessing autonomic nervous system activity. It has been associated with clinical outcomes across cardiovascular conditions and is extensively used in psychophysiological research on stress [2–5]. The time-domain and frequency-domain indices of HRV are regarded as standard measures and are codified in international consensus and reporting guidelines [5, 6].

Despite its potential, the translation of real-time HRV monitoring into widespread clinical practice has been hindered by fundamental methodological challenges. The first challenge is the frequent contamination of data by procedural artifacts inherent to the clinical environment, such as those arising from nursing care, patient movement, or emotion. These artifacts introduce non-physiological fluctuations into the RR intervals, distorting HRV calculations and compromising the reliability of the analysis [7]. While automated methods for evaluating signal quality have been proposed [7], they cannot fully account for the non-physiological fluctuations that occur in real-world clinical practice. Consequently, there is a critical need for a computational framework that can provide robust, personalized, and artifact-resilient HRV analysis.

The second challenge is the high inter-individual variability of HRV indices, which are significantly influenced by factors such as age and sex [8–11]. Conventional monitoring systems often rely on fixed, population-based thresholds for alerts, leading to a high rate of false positives or negatives and limiting their clinical utility [12]. Recent research has shown that personalized alarm thresholds, adapted to the statistical distribution of a patient’s own data, can effectively reduce non-actionable alarms while maintaining sensitivity to critical events [12, 13].

While several software tools for HRV analysis such as Kubios HRV are available and support various types of HRV indices, they are primarily designed for research and offline post hoc analysis rather than as clinical bedside monitoring systems [14–17]. Even though some tools provide real-time monitoring of HRV indices, they lack the capability for adaptive, personalized thresholding based on individual statistical distributions. Thus, a dedicated bedside solution that systematically integrates both personalization and artifact management for real-time monitoring remains lacking.

To address these gaps, we propose a new framework for real-time HRV monitoring designed to overcome these challenges. Our approach uniquely integrates adaptive, personalized analytics with a practical, workflow-integrated mechanism for artifact management. The core of our methodology consists of four key components: (1) an adaptive alerting algorithm that dynamically calculates patient-specific thresholds based on the statistical distribution (i.e., interquartile range) of their own whole data, (2) a workflow-integrated mechanism for manual annotation and exclusion of artifact-prone periods to enhance the robustness of the analysis, (3) the simultaneous analysis and visualization of both time- and frequency-domain indices to provide a comprehensive view of autonomic function, and (4) a multi-scale visualization approach that integrates short-term fluctuations with long-term trends to facilitate a more comprehensive clinical interpretation. We implemented this framework in a cross-platform software tool, named CODO Monitor, to demonstrate its feasibility and effectiveness. This paper details the proposed methodology and demonstrates its functional utility and potential clinical impact through a case study, presenting it as a new platform for advancing both patient care and clinical research in HRV.

## Methods

### System Implementation and Data Processing Pipeline

The proposed framework was implemented as a standalone executable application, CODO Monitor, using MATLAB AppDesigner and compiled for distribution. The distributed package includes the required MATLAB Runtime, enabling cross-platform use (Windows, macOS) without a MATLAB license or a separate MATLAB Runtime installation.

**Fig. 1:**
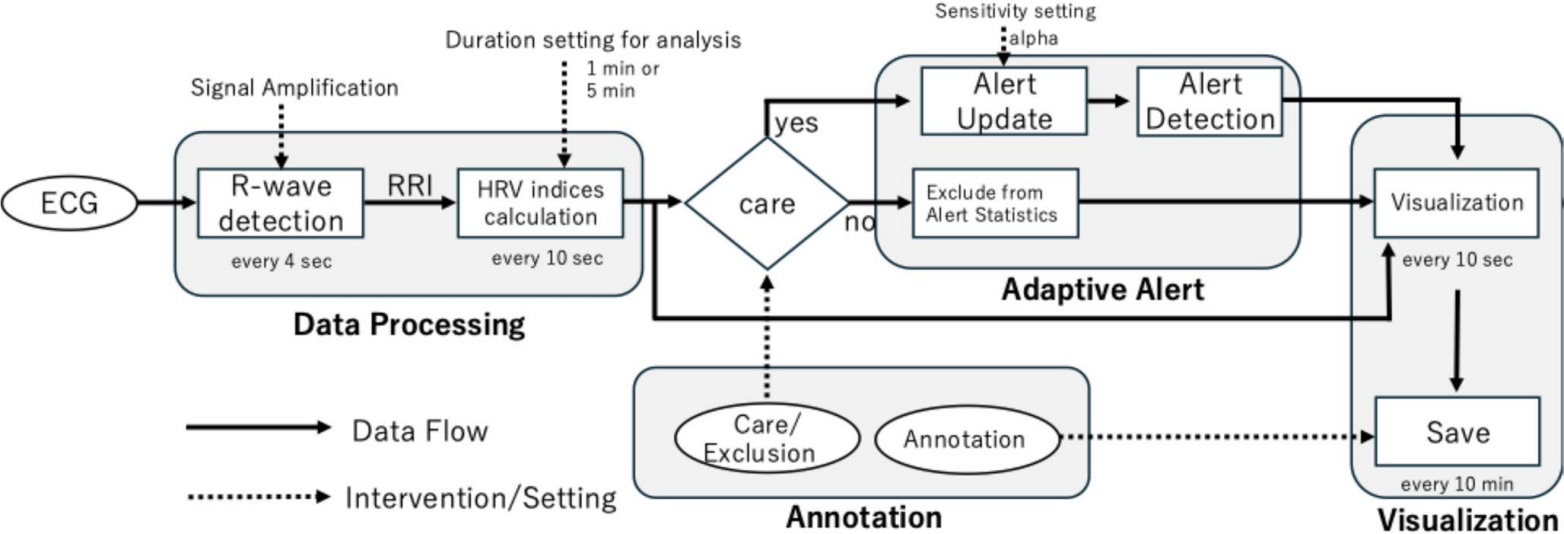
Flowchart of the clinically oriented real-time HRV framework. End-to-end workflow/data flow and interactions among the four core components 1 Data Processing, 2 Adaptive Alert, 3 Annotation, and 4 multi-scale visualization. Solid lines denote data flow; dashed lines denote user/feedback interactions

The workflow is demonstrated in Fig. 1. The data processing pipeline begins with the acquisition of an analog ECG signal, which is digitized at a sampling rate of 1000 Hz. To ensure stable R-wave peak detection, we simplified the Pan-Tompkins algorithm, including band-pass filtering, differentiation, non-linear transformation, and adaptive thresholding to identify QRS complexes in real time [18]. The detected R-wave peaks are used to calculate a continuous series of RR intervals (RRI). From this RRI time-series, a set of standard time-domain and frequency-domain HRV indices are computed every ten seconds using a moving window of the most recent one or five minutes of data. Time-domain indices include SDNN, RMSSD, pNN50, and non-linear indices of Poincaré plots (SD1, SD2, and SD1/SD2), while frequency-domain indices (VLF, LF, HF, and their ratios) are derived from a power spectral density analysis using the maximum entropy method [5, 6, 19, 20].

### Adaptive Alert Algorithm for Personalized Monitoring

To address the challenge of high inter-individual variability, we developed and implemented an adaptive alert algorithm. This method dynamically establishes a patient-specific normal range for each HRV index, rather than relying on fixed, population-based thresholds. The algorithm defines the upper and lower thresholds based on the statistical distribution of the patient’s own accumulated data. Specifically, the thresholds are calculated using the following formulas based on the interquartile range (IQR):

- Low threshold = Q1 - α(Q3 - Q1).
- High threshold = Q3 + α(Q3 - Q1).

Here, Q1 and Q3 represent the first and third quartiles of the patient’s data for a given index. The parameter α is a user-adjustable coefficient that modifies the sensitivity for outlier detection. In the present implementation, α can be selected from three discrete levels (α = 1.724, 2.465, and 3.207). Under the assumption of a Gaussian distribution with standard deviation σ, these values correspond approximately to thresholds at 3σ, 4σ, and 5σ from the mean, respectively. Thus, a lower α yields a more sensitive alerting behavior, whereas a higher α provides a more conservative threshold that reduces the number of alerts. When a newly calculated HRV index falls outside this dynamically updated range, an alert is triggered.

### Artifact Management Through Manual Annotation

To enhance the robustness of the analysis in the presence of procedural artifacts, we implemented a mechanism for manual event annotation during routine care. A user interface button allows clinicians to flag periods corresponding to nursing care, patient movement, or other events known to cause non-physiological signal fluctuations. Data points recorded during these annotated periods are visually distinguished in the output but are excluded from the dataset used to calculate the quartiles (Q1, Q3) for the adaptive alert algorithm. This exclusion prevents artifacts from skewing the statistical basis of the personalized thresholds, thereby improving the accuracy and reliability of the alert system. In the time-series plots, these annotated intervals are still displayed without any interpolation so that the temporal relationship between care procedures and physiological changes can be inspected, but they are rendered in a distinct color and excluded from the statistical computations used for adaptive threshold learning.

### Multi-Scale Visualization and Data Export of HRV Indices for Clinical Interpretation

To facilitate a comprehensive clinical assessment, the software provides an integrated, multi-scale visualization of HRV dynamics. The system is designed to simultaneously display time-series graphs of any selected combination of time- and frequency-domain indices, allowing for a comprehensive evaluation of autonomic function. Furthermore, these indices can be monitored across multiple, user-configurable time scales. This allows clinicians to monitor short-term, minute-by-minute fluctuations (reflecting immediate physiological responses) in parallel with long-term, multi-hour trends (reflecting slower changes in autonomic state), providing a more comprehensive overview of the patient’s condition. All calculated indices, alert events, and manual annotations are automatically logged and exported in standard formats (.xlsx and.mat) to facilitate offline analysis, clinical review, and further research.

### Functional Validation

Functional validation of the system consisted of two complementary steps. First, we quantified the accuracy and noise robustness of the R-wave detection module using open ECG databases. Recordings from the Fantasia Database and the Leipzig Heart Center ECG-Database [21, 22] were converted into WAV files and used as audio inputs to the system so that they would pass through the same software signal processing pipeline. The R-wave timings detected by the proposed system were compared with the reference annotations distributed with each database. To investigate robustness to noise, we generated synthetic baseline wander associated with respiration and added Gaussian noise to the original ECG signals based on [23]. The noise level was adjusted to obtain target signal-to-noise ratios (SNRs) of 3 dB and 6 dB. An R-wave detection was considered correct when the difference between the detected and annotated R-wave timing was within 50 ms. Detection performance was summarized in terms of sensitivity (true positives/(true positives + false negatives)) and precision (true positives/(true positives + false positives)). Second, we evaluated the overall functionality and bedside usability of the framework using ECG data obtained from 24 newborn patients monitored at Fujita Health University, under a protocol approved by the institutional ethics committee (approval number: CI24-1229) (Supplemental Table 1).

## Results

To validate the proposed framework, we applied the CODO Monitor software to analyze ECG data from newborn patients. The following sections demonstrate the performance and utility of the key methodologies implemented in the system.

### Reliability of the ECG Processing

**Fig. 2:**
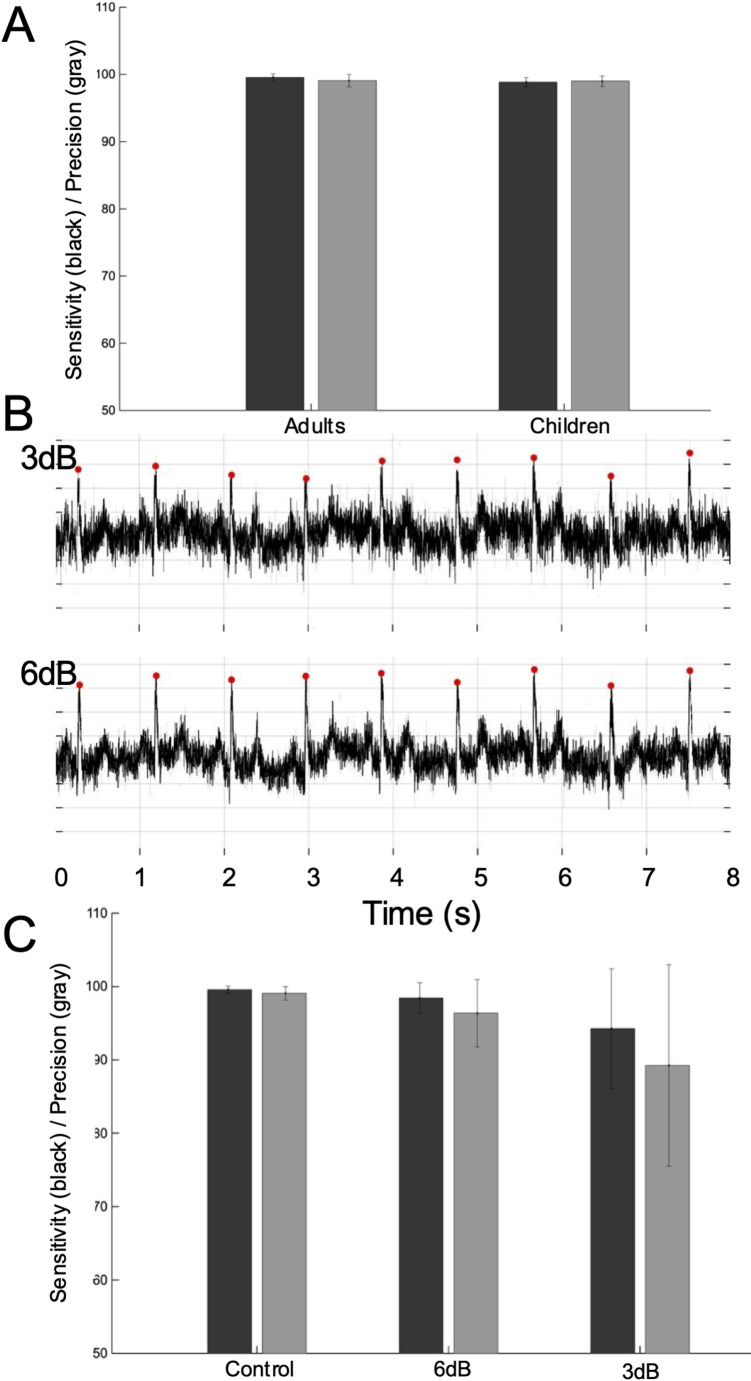
The reliability of R-wave detection. Panel A shows the sensitivity (black) and precision (gray) of R-wave detection compared with manual annotation using open datasets (Adults: Fantasia Database, Children: Leipzig Heart Center ECG-Database). Panel B shows the noise-added ECG signals from adults. The noise level is set so that the signal-to-noise ratio is 3 dB and 6 dB. The red points indicate the detected R-waves. Panel C shows the sensitivity (black) and precision (gray) of R-wave detection corresponding to different signal-to-noise ratio of 3 dB and 6 dB

The accuracy of the analysis heavily depends on the quality of the input ECG signal and the reliability of R-wave detection. We therefore quantified the performance of the system on open ECG datasets (Fig. 2). For adult recordings from the Fantasia Database and pediatric recordings from the Leipzig Heart Center ECG-Database, the proposed method achieved sensitivities of approximately 99.6% and 98.9%, and precisions of 99.1% and 99.0%, respectively. These values are comparable to those reported for other state-of-the-art R-wave detection algorithms [24].

To evaluate robustness to noise, we added synthetic baseline wander and Gaussian noise corresponding to SNRs of 6 dB and 3 dB to the original ECG signals. Even under these adverse conditions, sensitivity and precision remained 98.4% and 96.4% (6 dB), and 94.3% and 89.2% (3 dB), indicating that the detector maintains acceptable performance in the presence of substantial noise and is suitable for clinical environments where motion and electrical interference are common.

### Validation of the Adaptive Alert Algorithm

**Fig. 3:**
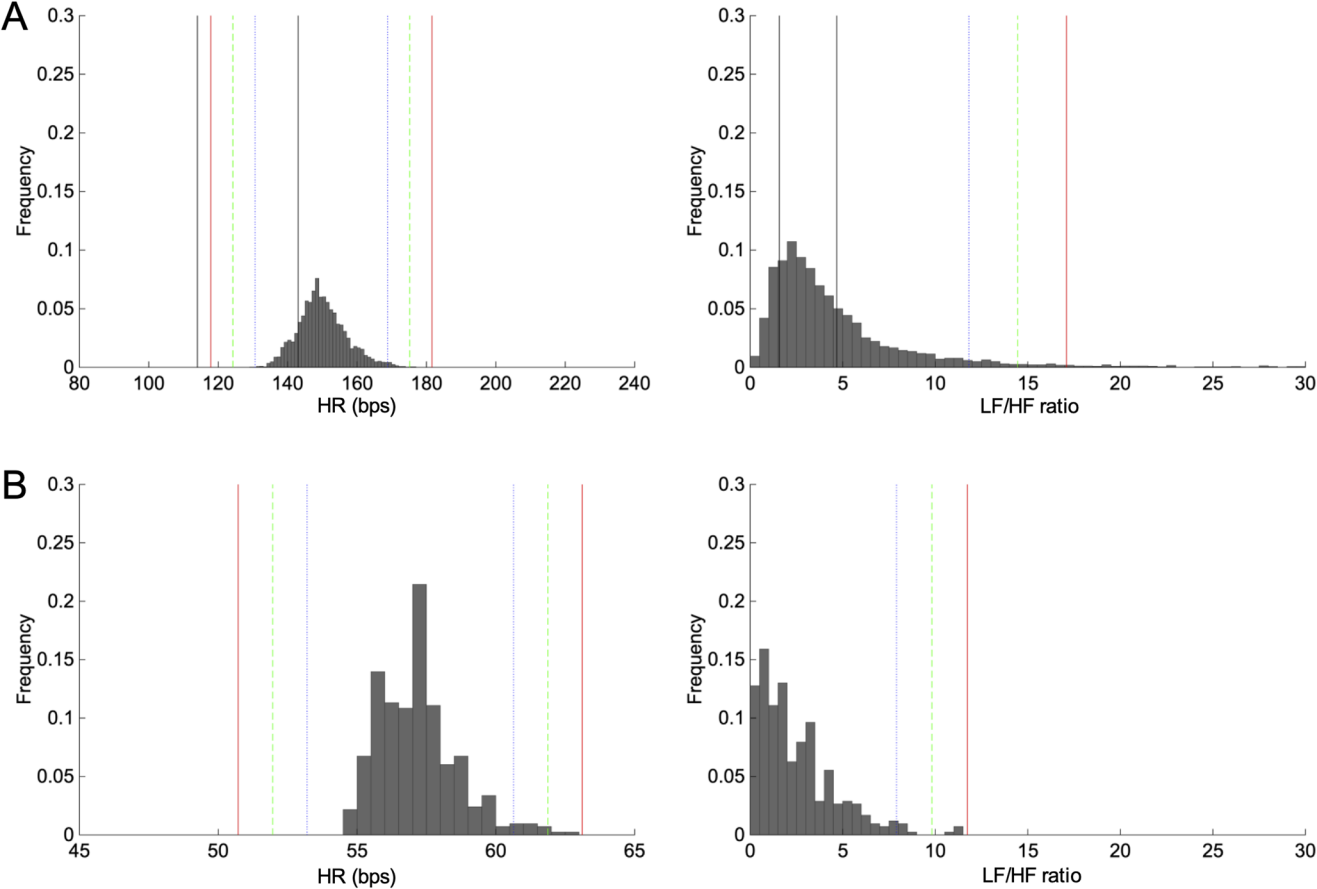
Demonstrations of distribution of HRV indices and adaptive threshold. Panel A shows HRV indices from a representative neonate patient (left: HR, right; LF/HF ratio). The blue dotted, green dashed and red solid lines indicate adaptive thresholds. The black lines indicate the 5th and 95 percentiles of HRV indices of newborn infants reported in [10]. Panel B shows HRV indices of an adult subject from open data [21]

The effectiveness of the personalized alert algorithm was assessed by comparing its dynamically generated thresholds against standard statistical values (mean and standard deviation). Figure 3 shows the frequency distribution of Heart Rate (HR) and the LF/HF ratio from a newborn patient and a subject from an open dataset. The patient’s HR distribution (left panel) was shifted relative to the reported normal range for newborn infants [10], illustrating how fixed, population-based thresholds could misclassify physiologically appropriate values as abnormal. Furthermore, the distribution of the LF/HF ratio (right panel) exhibited significant positive skew, a non-Gaussian characteristic commonly observed in HRV data. Under these conditions, thresholds derived from the IQR captured extreme values more appropriately than limits based on the mean and standard deviation, which assume approximate normality. Because the algorithm estimates thresholds from each individual’s empirical distribution, the same implementation could be applied without modification to adult data from open databases (Fig. 3B), despite substantial differences in HRV characteristics between newborns and adults. These findings indicate that the adaptive algorithm can personalize the normal range of HRV indices across diverse physiological states and data distributions.

### Efficacy of Artifact Management Through Manual Annotation

**Fig. 4:**
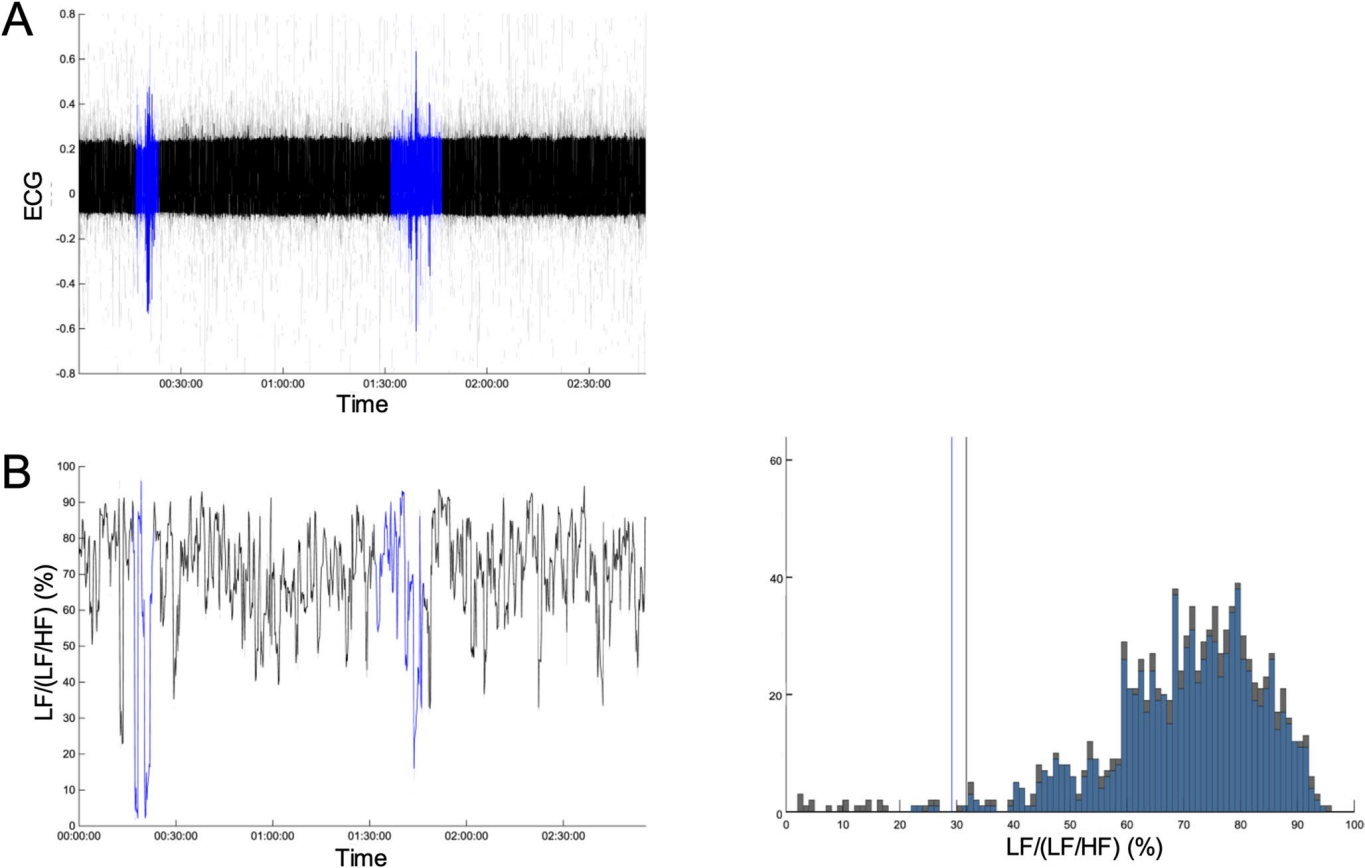
Demonstration of the exclusion by “Care/Data Exclusion” button. A timecourse of ECG signal is shown in panel A. The care period is highlighted in blue. Panel B shows the time course and histogram of LF/(LF + HF). The left panels show the timecourse, with blue indicating care. The right panels show histogram and lower adaptive alert threshold for the HRV index, with data during care shown in blue and data outside care shown in black

The clinical utility of the artifact management methodology is demonstrated in Fig. 4. A period of nursing care, manually annotated using the “Care/Data Exclusion” button, is highlighted in blue. During this period, the raw ECG signal (Panel A) shows significant noise, leading to large, non-physiological spikes in HRV indices such as RMSSD (Panel C, left). These artifactual data points distort the overall statistical distribution of the index. The right panels of B and C show the histograms of LF/(LF + HF) and RMSSD, respectively, with and without the annotated data. By excluding the artifactual data (blue bars) from the statistical calculation, the adaptive alert threshold (vertical line) is based only on the patient’s underlying physiological state (grey bars). This result demonstrates that the manual annotation and exclusion process is effective in preventing procedural artifacts from corrupting the personalized thresholds, thereby enhancing the reliability and accuracy of the alert system.

### Utility of Multi-Scale Visualization for Clinical Interpretation

**Fig. 5:**
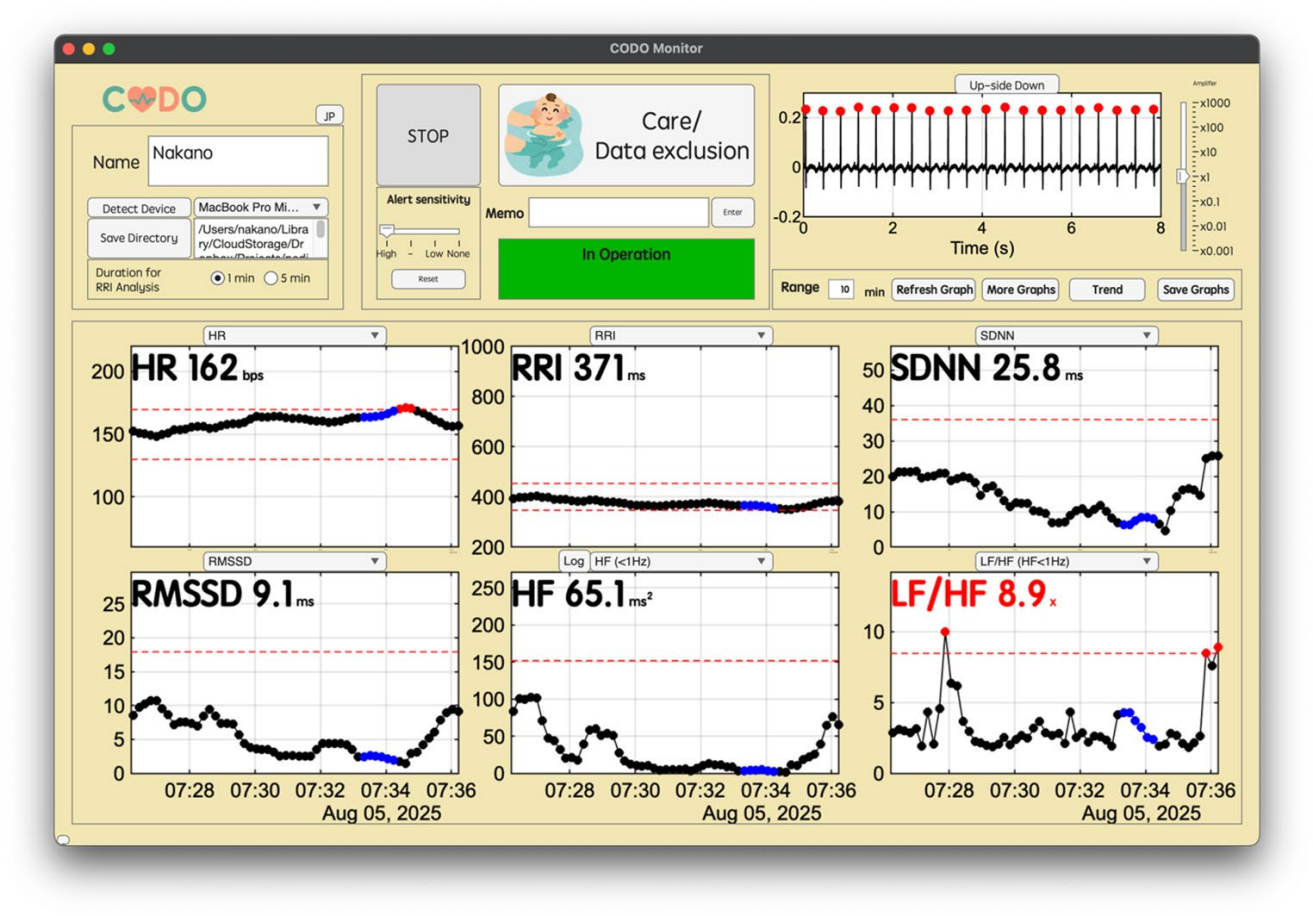
The main window of CODO Monitor. The upper panel contains settings and control buttons. The top-right graph displays the real-time ECG waveform with detected R-wave peaks (red dots). The six lower graphs show time-series data for selected HRV indices. Outliers exceeding personalized thresholds are highlighted in red and drawn with dashed red lines. Data points recorded during periods marked by the “Care/Data Exclusion” button are shown in blue

**Fig. 6:**
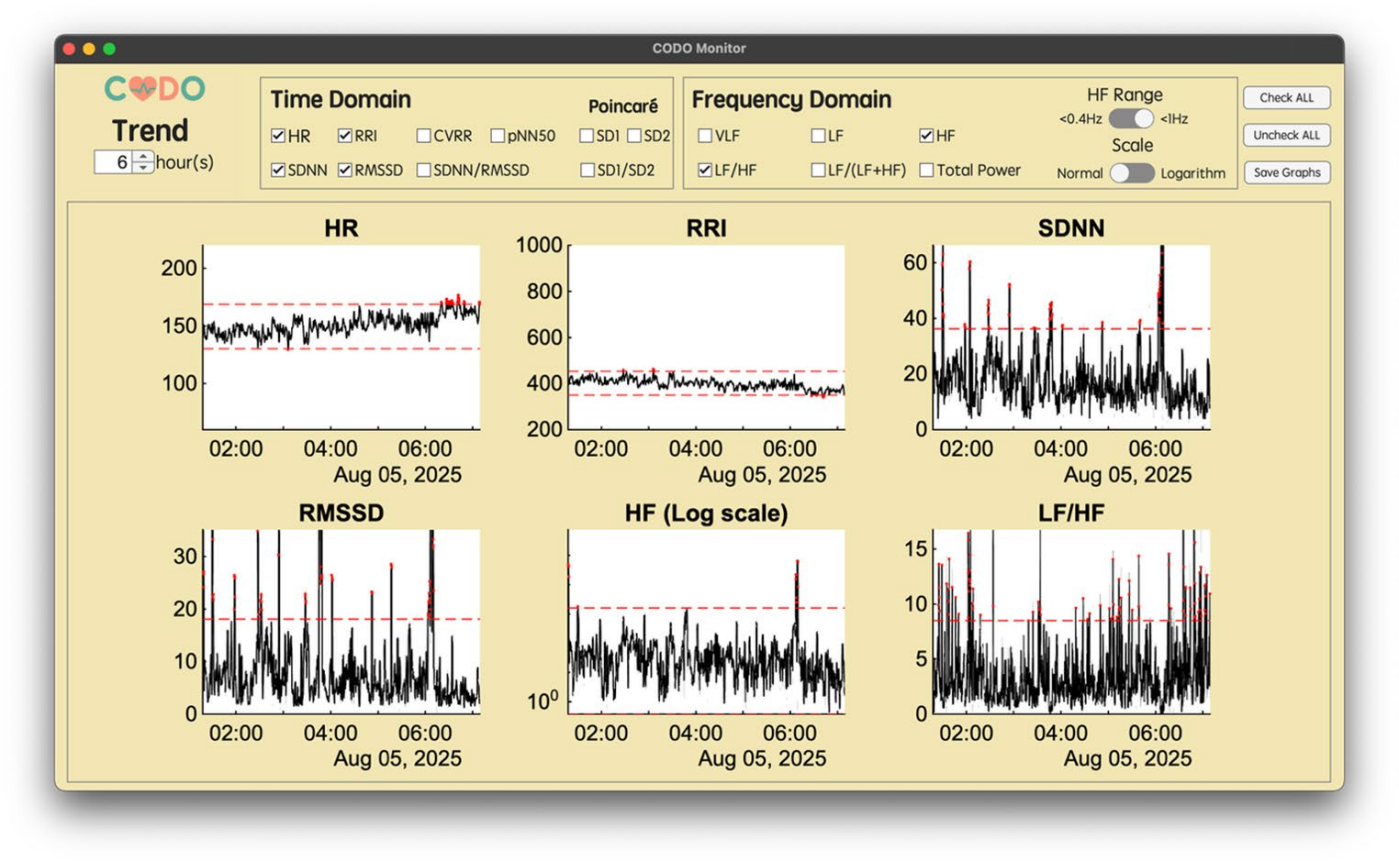
The “Trend” window for long-term data visualization. This view displays long-term trends of selected HRV indices over a user-defined period, such as multiple hours. It provides a comprehensive overview of a patient’s condition by showing slow changes over time with the personalized normal range (indicated by red dashed lines)

**Fig. 7:**
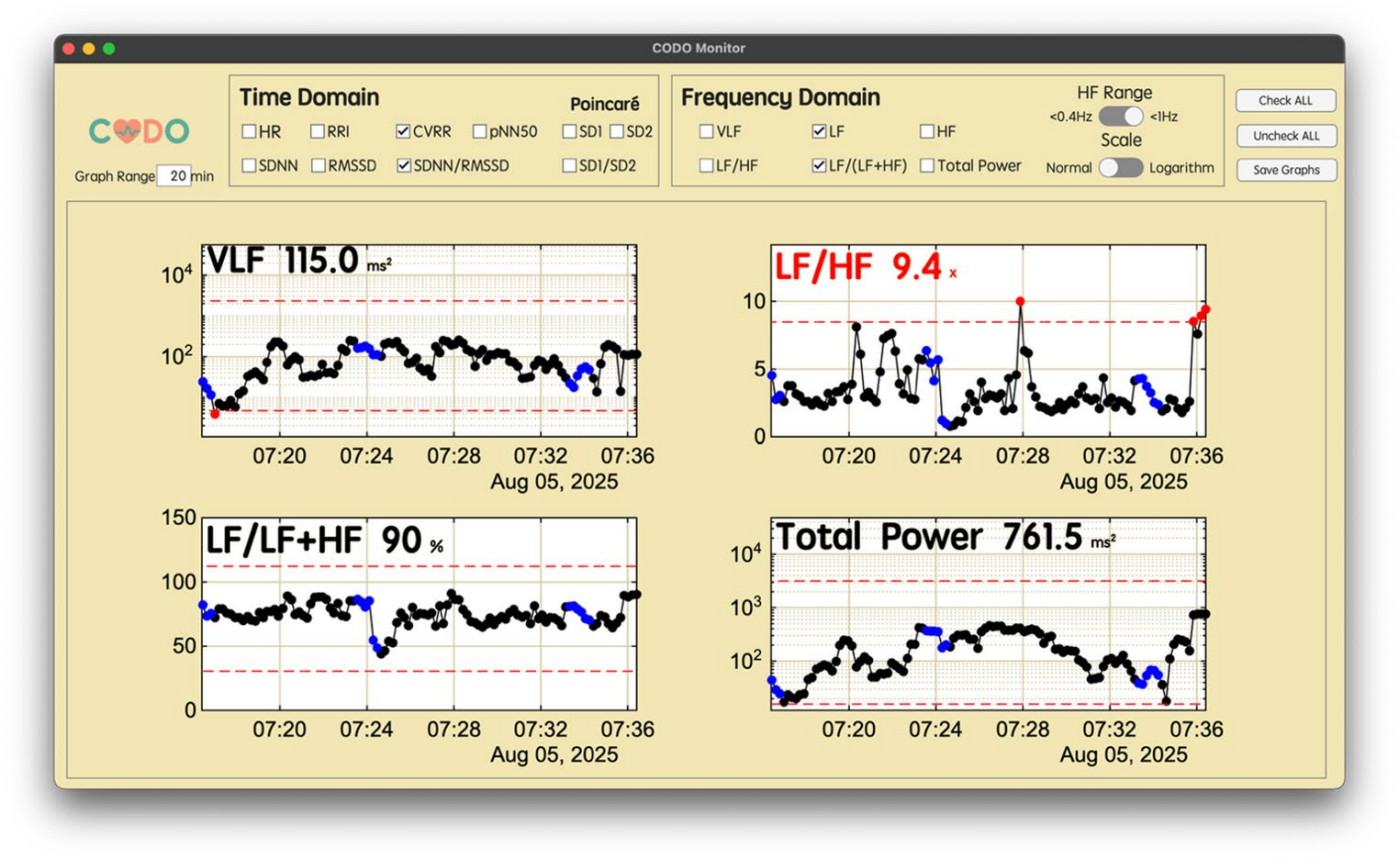
The “More Graphs” window. This window, accessible via the “More Graphs” button on the main window, allows the user to simultaneously display a larger set of HRV indices than the main window. Users can customize the view by selecting specific time-domain and frequency-domain indices using the checkboxes at the top. It also provides options to adjust the calculation range for High Frequency (HF) and to switch the display to a logarithmic scale for some frequency domain indices

The framework’s real-time output is presented through a graphical user interface that implements the multi-scale visualization methodology (Fig. 5). The main window displays a user-selected combination of time- and frequency-domain HRV indices, enabling a comprehensive assessment of autonomic function on a short time scale (e.g., 1 to 60 min). This allows for the immediate detection of rapid changes and outliers highlighted by the adaptive alert system. To provide clinical context to these short-term events, the system can simultaneously display long-term trend graphs of the same indices over several hours (Fig. 6). This dual-scale view enables clinicians to distinguish transient fluctuations from sustained shifts in autonomic state, facilitating a more comprehensive and informed interpretation of the patient’s condition. The interface, as shown in Figs. 5 and 6, and 7, serves as the platform for visualizing the outputs of the integrated analytical pipeline.

### Structured Data Export for Offline Analysis

The system automatically generates structured data files in.xlsx format, systematically logging all computed HRV indices, timestamps, alert occurrences, and manual annotations (Supplemental Tables 2–4). This automated export function ensures that all data and metadata are captured in a format amenable to subsequent offline analysis, supporting clinical review and further research activities.

## Discussion

In this study, we developed and validated a novel computational framework for real-time HRV analysis, implemented in the software tool CODO Monitor. Our work addresses two of the most significant barriers to the clinical application of real-time HRV monitoring: high inter-individual variability and data contamination from procedural artifacts. The proposed methodologies—an adaptive alert algorithm, a manual artifact management system, and a multi-scale visualization approach—collectively enhance the robustness, personalization, and interpretability of HRV analysis in complex clinical environments.

A key contribution of our framework is the adaptive alert algorithm, which personalizes alert thresholds using the statistical distribution of each patient’s own data. As demonstrated in our results, physiological indices such as HR can vary significantly from population norms, and HRV metrics often exhibit non-Gaussian distributions. By employing an IQR-based method, our algorithm provides a statistically robust approach that accommodates these individual-specific and distributional characteristics. In practical terms, the three preset levels of α in our implementation can be interpreted as ‘sensitive’ (α = 1.724), ‘intermediate’ (α = 2.465), and ‘conservative’ (α = 3.207) alarm strategies. In our case-study dataset, decreasing α from 3.207 to 1.724 increased the fraction of alerts from 2.7% to 6.2% for the LF/HF ratio, illustrating the expected trade-off between sensitivity and the number of alerts. In clinical practice, α can therefore be chosen according to the desired balance between early detection of subtle changes and avoidance of alarm fatigue. This methodology represents a substantial improvement over conventional fixed-threshold systems, offering the potential to increase the specificity of alerts, reduce alert fatigue among clinical staff, and ultimately make real-time monitoring more clinically meaningful.

Furthermore, the manual annotation and exclusion mechanism directly confronts the challenge of data quality in real-world clinical settings. Procedural artifacts are an unavoidable reality of patient care, yet they can severely compromise the validity of HRV indices. Our results confirm that by enabling clinicians to exclude these contaminated periods from statistical calculations, the reliability of the personalized thresholds is significantly improved. This workflow-integrated approach to data cleaning is a practical and effective method for enhancing the robustness of real-time analysis, ensuring that clinical decisions are based on the patient’s underlying physiological state rather than on noisy, artifactual data.

The integration of these components within a multi-scale visualization platform transforms the system from a simple data monitor into a powerful tool for clinical interpretation and research. This is particularly significant in neonatal care. ECGs are noninvasive and provide continuous, real-time data, and previous studies have reported an association between autonomic dysfunction and severe infections in infants [25]. However, analyzing ECGs from very preterm infants has been challenging due to the difficulty in obtaining stable data amidst frequent nursing care and crying. Our framework addresses this specific challenge. The ability to reliably assess cardiac autonomic activity by excluding artifact-prone periods from noninvasive bedside monitor signals provides an extremely useful source of information for future neonatal care. Our framework was operationally validated at the bedside using ECG data from 24 newborn patients. This approach, enabling robust analysis even in a noisy clinical environment, is a major step forward in making real-time autonomic assessment a clinical reality.

This study has several limitations. First, the accuracy of the entire analysis pipeline is fundamentally dependent on the quality of the input ECG signal and the reliability of R-wave detection. There are various methods for R-wave detection, and this study did not conduct a comprehensive comparative analysis. Exploring faster and more accurate methods remains a future challenge. However, as shown in Fig. 2, the current system handles both pediatric and adult data and demonstrates performance comparable to other R-wave detection methods. It also successfully detects R-waves in noisy conditions. Furthermore, data have been collected from 24 newborns, and experts in neonatal electrocardiography have reported no issues with the R-wave detection. While validated with such clinical data, the algorithm’s performance under conditions of extreme noise has not been exhaustively tested. Future work should focus on enhancing the signal processing front-end to improve robustness.

Second, the current implementation utilizes an analog signal input. While digital integration offers seamless data transfer, it is often hindered by manufacturer-specific proprietary protocols that restrict interoperability. By leveraging the standard analog output available on most major monitoring systems (e.g., Philips, GE Healthcare, Nihon Kohden), our framework achieves universal compatibility without requiring expensive, vendor-specific middleware. This design choice prioritizes immediate clinical applicability and accessibility across diverse hospital environments. While the analog approach ensures broad compatibility, future expansion to support direct digital data streams from hospital networks would further enhance integration capabilities.

Third, the care/data exclusion function and manual annotations of artifacts introduced in this system allow doctors and nurses to annotate during care and procedures, making it particularly useful for neonatal care with minimal burden. Because these annotations are time-locked to concrete clinical events, they serve a dual purpose: they define which periods should be excluded from learning the statistical baseline, and they also provide a structured record that links interventions (e.g., repositioning, suction, medication) to the subsequent evolution of HRV indices. This dual role is clinically important when interpreting whether a sudden change in autonomic activity is plausibly explained by ongoing care or may instead indicate emerging pathology. For this reason, we deliberately avoided purely automatic artifact rejection based solely on signal-quality metrics or waveform morphology. By restricting exclusion to periods that staff explicitly mark as care-related, the framework minimizes the risk of inadvertently removing genuine physiological instability while still protecting the adaptive thresholds from obvious procedural artifacts. However, this strategy requires staff intervention and cannot eliminate all non-care-related artifacts. Even though quartile-based statistics are less affected by outliers, explicitly removing artifacts further improves overall data quality. In particular, spontaneous crying, motion, or technical issues still need to be identified by staff, which can be burdensome in a busy NICU. Future work will be integrating automatic signal-quality indices and machine-learning–based suggestions for care/noise annotation to reduce this burden while preserving clinician control over which data are removed from threshold learning and clinical interpretation.

Several frameworks and software packages have been developed for HRV analysis. Representative research-oriented tools include Kubios HRV, RapidHRV, RR-APET, and the open-source spectral analysis toolbox by Rodríguez-Liñares et al. [17]. These packages support a wide range of time-, frequency-, and in some cases non-linear indices. CODO Monitor currently supports the following time-domain indices: pNN50, SDNN, RMSSD, and the SDNN/RMSSD ratio, non-linear indices of Poincaré plots: SD1, SD2, and SD1/SD2, and frequency-domain indices: VLF, LF, HF, and their ratios. The number of supported HRV indices is lower than that of Kubios HRV [14], widely used for HRV analysis, providing a broad set of time-, frequency-, and non-linear indices. Future work will include supporting other indices, including Higuchi fractal dimension, acceleration capacity and deceleration capacity.

On the other hand, most of systems support only offline post-hoc analysis. They are lacking real-time processing pipelines, explicit artifact-management mechanisms, and workflow-integrated user interfaces tailored to continuous bedside monitoring and clinical alert management. Systems that support monitoring HRV indices during acquisition include the mobile version of Kubios HRV, MemCalc, LabChart HRV, AcqKnowledge HRV, and OpenSignals. Kubios HRV mobile app offers real-time visualization and artifact correction. Nevertheless, its alert functions mainly target signal-quality issues rather than implementing personalized alarm algorithms that automatically optimize thresholds based on each patient’s statistical baseline. LabChart with HRV Module (ADInstruments, Dunedin, New Zealand), AcqKnowledge software (BIOPAC Systems, Inc., Goleta, CA, USA), and OpenSignals (PLUX Wireless Biosignals S.A., Arruda dos Vinhos, Portugal) are versatile physiological recording systems used with dedicated hardware. They either do not provide HRV-based alerting or, do not include an adaptive threshold. MemCalc/Win (GMS Co., Ltd., Tokyo, Japan) [26] supports real-time monitoring of time- and frequency-domain indices and has a track record of clinical use, yet it does not incorporate automatically adaptive, patient-specific thresholds or an artifact-management mechanism that is linked to manual annotations. In comparison, the proposed CODO Monitor uniquely integrates, within a single stand-alone software package, (i) an IQR-based adaptive threshold-learning algorithm that personalizes alerts for each HRV index using the patient’s own distribution, (ii) a workflow-integrated artifact-management scheme in which bedside procedures (e.g., nursing care) can be annotated with a single click and automatically excluded from the statistical computations in real time, and (iii) a multi-scale visualization module that simultaneously displays short-term (minutes) and long-term (hours) HRV dynamics. Thus, CODO Monitor is positioned as a “clinically integrated bedside solution for personalized and artifact-resilient real-time HRV monitoring,” filling a gap that is not addressed by existing open-source or commercial HRV tools.

Finally, while this paper establishes the technical and functional validity of our framework, large-scale clinical studies are required to verify its effectiveness in improving patient outcomes. Such studies remain a crucial objective for future research.

In conclusion, this paper presents a clinically-oriented framework that improves the practicality and reliability of real-time HRV monitoring. By systematically addressing the core challenges of personalization and artifact management, our methodology provides a robust platform for both clinical care and research. This work represents a significant step toward translating the potential of real-time HRV analysis into tangible improvements in vital sign management and patient outcomes.

## Supplementary Information

Supplementary Material 1

## Data Availability

No datasets were generated or analysed during the current study.

## References

[1] Moorman JR, Carlo WA, Kattwinkel J, et al (2011) Mortality Reduction by Heart Rate Characteristic Monitoring in Very Low Birth Weight Neonates: A Randomized Trial. J Pediatr 159:900–906.e1. 10.1016/j.jpeds.2011.06.044 21864846 10.1016/j.jpeds.2011.06.044 PMC3215822

[2] Pizarro C, Bosse FL, Begrich C, et al (2023) Cardiac autonomic dysfunction in adult congenital heart disease. BMC Cardiovasc Disord 23:513. 10.1186/s12872-023-03558-4 37864159 10.1186/s12872-023-03558-4 PMC10589992

[3] Toyofuku A, Ehrler M, Naef N, et al (2025) Heart rate variability and cognitive functions in adolescents with complex congenital heart disease. Pediatr Res 97:1103–1113. 10.1038/s41390-024-03432-9 39080463 10.1038/s41390-024-03432-9 PMC12055568

[4] Westhoff-Bleck M, Lemke LH, Bleck J-MS, et al (2021) Depression Associated with Reduced Heart Rate Variability Predicts Outcome in Adult Congenital Heart Disease. J Clin Med 10:1554. 10.3390/jcm10081554 33917168 10.3390/jcm10081554 PMC8067842

[5] Quigley KS, Gianaros PJ, Norman GJ, et al (2024) Publication guidelines for human heart rate and heart rate variability studies in psychophysiology—Part 1: Physiological underpinnings and foundations of measurement. Psychophysiology 61:e14604–e14604. 10.1111/psyp.14604 38873876 10.1111/psyp.14604 PMC11539922

[6] Sassi R, Cerutti S, Lombardi F, et al (2015) Advances in heart rate variability signal analysis: joint position statement by the e-Cardiology ESC Working Group and the European Heart Rhythm Association co-endorsed by the Asia Pacific Heart Rhythm Society. EP Eur 17:1341–1353. 10.1093/europace/euv015 10.1093/europace/euv015 26177817

[7] Orphanidou C, Bonnici T, Charlton P, et al (2015) Signal-Quality Indices for the Electrocardiogram and Photoplethysmogram: Derivation and Applications to Wireless Monitoring. IEEE J Biomed Heal Inform 19:832–838. 10.1109/jbhi.2014.2338351 10.1109/JBHI.2014.2338351 25069129

[8] Gąsior JS, Sacha J, Pawłowski M, et al (2018) Normative Values for Heart Rate Variability Parameters in School-Aged Children: Simple Approach Considering Differences in Average Heart Rate. Front Physiol 9:1495. 10.3389/fphys.2018.01495 30405445 10.3389/fphys.2018.01495 PMC6207594

[9] Sharma VK, Subramanian SK, Arunachalam V, Rajendran R (2015) Heart Rate Variability in Adolescents – Normative Data Stratified by Sex and Physical Activity. J Clin Diagn Res 9:CC08-13. 10.7860/jcdr/2015/15373.6662 26557514 10.7860/JCDR/2015/15373.6662 PMC4625233

[10] Mehta SK, Super DM, Connuck D, et al (2002) Heart rate variability in healthy newborn infants. Am J Cardiol 89:50–53. 10.1016/s0002-9149(01)02162-2 11779522 10.1016/s0002-9149(01)02162-2

[11] Damoun N, Amekran Y, Taiek N, Hangouche AJE (2024) Heart rate variability measurement and influencing factors: Towards the standardization of methodology. Glob Cardiol Sci Pr 2024:e202435. 10.21542/gcsp.2024.35 10.21542/gcsp.2024.35 PMC11439429 39351472

[12] Rossum MC van, Vlaskamp LB, Posthuma LM, et al (2022) Adaptive threshold-based alarm strategies for continuous vital signs monitoring. J Clin Monit Comput 36:407–417. 10.1007/s10877-021-00666-4 33575922 10.1007/s10877-021-00666-4 PMC9123069

[13] Poole S, Shah N (2017) Addressing vital sign alarm fatigue using personalized alarm thresholds. Pac Symp Biocomput Pac Symp Biocomput 23:472–483 PMC6587573 29218906

[14] Tarvainen MP, Niskanen J-P, Lipponen JA, et al (2014) Kubios HRV – Heart rate variability analysis software. Comput Methods Programs Biomed 113:210–220. 10.1016/j.cmpb.2013.07.024 24054542 10.1016/j.cmpb.2013.07.024

[15] Kirk PA, Bryan AD, Garfinkel SN, Robinson OJ (2022) RapidHRV: an open-source toolbox for extracting heart rate and heart rate variability. PeerJ 10:e13147. 10.7717/peerj.13147 35345583 10.7717/peerj.13147 PMC8957280

[16] McConnell M, Schwerin B, So S, Richards B (2020) RR-APET - Heart rate variability analysis software. Comput Methods Programs Biomed 185:105127. 10.1016/j.cmpb.2019.105127 31648100 10.1016/j.cmpb.2019.105127

[17] Rodríguez-Liñares L, Méndez AJ, Lado MJ, et al (2011) An open source tool for heart rate variability spectral analysis. Comput Methods Programs Biomed 103:39–50. 10.1016/j.cmpb.2010.05.012 20674067 10.1016/j.cmpb.2010.05.012

[18] Pan J, Tompkins WJ (1985) A Real-Time QRS Detection Algorithm. IEEE Trans Biomed Eng BME-32:230–236. 10.1109/tbme.1985.325532 10.1109/TBME.1985.325532 3997178

[19] Task Force of The European Society of Cardiology and The North American Society of Pacing and Electrophysiology (1996) Heart rate variability: standards of measurement, physiological interpretation and clinical use. European Heart Journal 17:354–381 8737210

[20] Kay SM, Jr. SLM (1981) Spectrum analysis—A modern perspective. Proc IEEE 69:1380–1419.10.1109/proc.1981.12184

[21] Iyengar N, Peng CK, Morin R, et al (1996) Age-related alterations in the fractal scaling of cardiac interbeat interval dynamics. Am J Physiol-Regul, Integr Comp Physiol 271:R1078–R1084. 10.1152/ajpregu.1996.271.4.r1078 10.1152/ajpregu.1996.271.4.R1078 8898003

[22] Sophia K, Daniel F, Bayhas A, et al (2025) Leipzig Heart Center ECG-Database: Arrhythmias in Children and Patients with Congenital Heart Disease. PhysioNet. 10.13026/7a4j-vn37

[23] McSharry PE, Clifford GD, Tarassenko L, Smith LA (2003) A dynamical model for generating synthetic electrocardiogram signals. IEEE Trans Biomed Eng 50:289–294. 10.1109/tbme.2003.808805 12669985 10.1109/TBME.2003.808805

[24] Elgendi M (2013) Fast QRS Detection with an Optimized Knowledge-Based Method: Evaluation on 11 Standard ECG Databases. PLoS ONE 8:e73557. 10.1371/journal.pone.0073557 24066054 10.1371/journal.pone.0073557 PMC3774726

[25] Sullivan BA, McClure C, Hicks J, et al (2016) Early Heart Rate Characteristics Predict Death and Morbidities in Preterm Infants. J Pediatr 174:57–62. 10.1016/j.jpeds.2016.03.042 27113378 10.1016/j.jpeds.2016.03.042 PMC5672906

[26] Kasaoka S, Nakahara T, Kawamura Y, et al (2010) Real-time monitoring of heart rate variability in critically ill patients. J Crit Care 25:313–316. 10.1016/j.jcrc.2009.06.047 19781905 10.1016/j.jcrc.2009.06.047

