# Supplementary material for: A Clinically Oriented Framework for Real-Time Heart Rate Variability Analysis: A Novel Approach To Personalized and Robust Monitoring
Source: J Med Syst. 2026 Jan 29;50(1):13. doi: 10.1007/s10916-026-02342-z (PMC12855435; doi:10.1007/s10916-026-02342-z)
Supplement: Supplementary file 1 [file 10916_2026_2342_MOESM1_ESM.docx]

Supplemental 

upplemental Table 1 Patient demographics

|  | n=24 |
| --- | --- |
| Gestational age at birth(weeks; median[IQR]) | 27 [25, 36] |
| Sex, Male (n [%]) | 13  [54.2] |
| Birth weight (g; median[IQR]) | 970 [716, 1352] |
| Recording Time (minutes; median [IQR]) | 10072 [5260, 19535] |
| Post natal day at recording start (days; median [IQR]) | 0 [0, 1] |
| Post natal day at recording completion (days; median [IQR]) | 9 [5, 14] |

Supplemental Table 2 Example excerpt of exported Excel log (2‑min interval)

| data_time | care | memo | RRI | HR | … | Total Power | Total Power (HF<1Hz) |
| --- | --- | --- | --- | --- | --- | --- | --- |
| 2025/6/25 12:56 | 0 | 0 | 430.26 | 139.46 | … | 237.14 | 250.53 |
| 2025/6/25 12:58 | 0 | 0 | 418.99 | 143.27 | … | 237.02 | 245.48 |
| 2025/6/25 13:00 | 1 | 0 | 429.37 | 139.81 | … | 381.95 | 402.84 |
| 2025/6/25 13:02 | 0 | 1 | 418.29 | 143.45 | … | 216.13 | 222.38 |
| 2025/6/25 13:04 | 0 | 0 | 419.39 | 143.07 | … | 1137.33 | 1172.32 |
| 2025/6/25 13:06 | 0 | 0 | 410.57 | 146.15 | … | 231.05 | 243.98 |
| 2025/6/25 13:08 | 1 | 0 | 413.07 | 145.28 | … | 155.61 | 158.66 |
| 2025/6/25 13:10 | 0 | 2 | 425.12 | 141.15 | … | 1820.33 | 2162.35 |
| 2025/6/25 13:12 | 0 | 0 | 429.41 | 139.77 | … | 596.48 | 610.64 |
| 2025/6/25 13:14 | 0 | 0 | 428.41 | 140.10 | … | 553.80 | 570.45 |
| 2025/6/25 13:16 | 0 | 0 | 433.52 | 138.41 | … | 521.57 | 537.13 |

Supplemental Table 3 Example of Memo sheet

| Index | Time | Memo |
| --- | --- | --- |
| 1 | 2025/6/25 13:00 | medication |
| 2 | 2025/6/25 13:09 | cry |

Supplemental Table 4 Example of Alert sheet

| Time | RRI_alert | … | RMSSD_alert | RRI | … | RMSSD |
| --- | --- | --- | --- | --- | --- | --- |
| 2025/6/25 13:09 | 0 | … | 1 | 426.871 | … | 67.729 |
| 2025/6/25 13:10 | 0 | … | 1 | 422.085 | … | 67.242 |
| 2025/6/25 13:10 | 0 | … | 1 | 421.723 | … | 67.265 |
| 2025/6/25 13:10 | 0 | … | 1 | 425.671 | … | 67.633 |
| 2025/6/25 13:12 | 0 | … | 0 | 421.979 | … | 26.341 |
| 2025/6/25 13:12 | 0 | … | 0 | 420.725 | … | 26.276 |
| 2025/6/25 13:13 | 0 | … | 0 | 417.706 | … | 26.072 |
| 2025/6/25 13:13 | 0 | … | 0 | 420.851 | … | 26.382 |
| 2025/6/25 13:13 | 0 | … | 0 | 425.85 | … | 26.554 |
| 2025/6/25 13:13 | 0 | … | 0 | 426.871 | … | 67.729 |
